# Female preferences for male traits and territory characteristics in the cichlid fish *Tropheus moorii*

**DOI:** 10.1007/s10750-014-1892-7

**Published:** 2015-04-01

**Authors:** Caroline M. Hermann, Verena Brudermann, Holger Zimmermann, Johann Vollmann, Kristina M. Sefc

**Affiliations:** Department of Zoology, University of Graz, Universitätsplatz 2, 8010 Graz, Austria; Department of Zoology, University of Graz, Universitätsplatz 2, 8010 Graz, Austria; Department of Zoology, University of Graz, Universitätsplatz 2, 8010 Graz, Austria; Division of Plant Breeding, Department of Crop Sciences, University of Natural Resources and Life Sciences, Vienna (BOKU), Konrad Lorenz Str. 24, 3430 Tulln, Austria; Department of Zoology, University of Graz, Universitätsplatz 2, 8010 Graz, Austria

**Keywords:** Mate choice, Territory quality, Territory size, Body size, Red color, Assortative mating

## Abstract

Female mate preferences for male traits and resource characteristics affect trait evolution and diversification. Here, we test the effects of male body traits and territory characteristics on within-population female preferences and on population–assortative mating in the cichlid *Tropheus moorii*. Within-population preferences of females were independent of male body size, coloration and territory size but were strongly dependent on territory quality and co-varied with male courtship activity. Courtship activity of individual males was contingent on the quality of their assigned territory, and therefore, courtship may not only indicate intrinsic male quality. On the basis of these results we suggest that female preferences for high-quality territories reinforce the outcome of malemale competition and ensure male mating success. Mating preferences of females for males of their own color variant (ascertained in a previous experiment) were not overturned when males of another color variant were presented in a superior territory, indicating that within- and between-population mate preferences of females depend on different cues.

## Introduction

In many animal species, females are expected to choose mates according to criteria that are relevant to their own fitness (Bluhm & Gowaty, 2004; Neff & Pitcher, 2005). Direct benefits obtained from adaptive mate choice include material resources provided by the male, as well as brood care and protection, which can increase the female’s present and lifetime fecundity. Indirect benefits for the female are derived from high fitness of her offspring, to which the male contributes genes and parental investment (Neff & Pitcher, 2005). Trade-offs between direct and indirect benefits have been identified in several species (Wong & Candolin, 2005), but positive correlations are also possible, for example, if the genetic quality of a male also benefits a female mate directly. If males require certain resources such as territories or nest sites for mating, male–male competition can narrow the pool of candidate mates to competitive individuals and thus can facilitate female choice (Wong & Candolin, 2005).

Female choice among males that succeed in intrasexual competition for resources may be based on characteristics of the males themselves, such as size, coloration, or behavior, as well as on the quality of their resources (Candolin, 2003). Females of some species, especially among fish, have been shown to prefer larger over smaller males (Poeciliidae: Ptacek & Travis, 1997; Rosenthal & Evans, 1998; Basolo, 2004; Cyprinidae: Hudman & Gotelli, 2007; Rivulidae: Passos et al., 2013; spiders: Schütz & Taborsky, 2011). Presumed benefits for females mated to large males include protection from harassment and predation, increased fertilization and heritable dominance status (Basolo, 2004; Hudman & Gotelli, 2007). Male coloration is another potential mate choice cue. In particular, female preferences for males with intensive red and orange coloration (birds: Hill et al., 1999; reptiles: Kwiatkowski & Sullivan, 2002; amphibians: Gomez et al., 2009; fish: Milinski & Bakker, 1990; Kodric-Brown, 1993; Maan et al., 2004) may be related to the connection between carotenoid pigmentation and individual condition (Svensson & Wong, 2011). Correlations between female preferences and male courtship effort are also widespread (e.g., wolf spiders, Shamble et al., 2009; fish, Kodric-Brown, 1993; Grant & Green, 1996; Forsgren, 1997; Amorim et al., 2013; skinks, Stapley, 2008; birds, Bossema & Kruijt, 1982), and there is some evidence that male courtship effort can signal male quality to females (Eckert & Weatherhead, 1987; Knapp & Kovach, 1991; Weir & Grant, 2010; Pedroso et al., 2013). Sound production is an important component of courtship displays in many animals, including insects, birds, and anurans (Searcy & Andersson, 1986). Males of fish species can also produce sounds during courtship displays (reviewed in Amorim, 2006), and mate choice experiments have confirmed the influence of fish courtship sounds on female preferences (McKibben & Bass, 1998; Verzijden et al., 2010; Maruska et al., 2012).

In species, in which male resources either provide direct benefits to female fitness or serve as indicators of male quality, resource characteristics frequently influence female choice (birds: Alatalo et al., 1986; Eckerle & Thompson, 2006; Hasegawa et al., 2012; fish: Candolin & Reynolds, 2001; Maan et al., 2004; Lehtonen et al., 2007; Dijkstra et al., 2008; Schaedelin & Taborsky, 2010). Using territory quality as a mate choice cue, females may profit from shelter and food provided by the territory, and at the same time mate with a male in good condition, if male–male competition restricts ownership of high-quality territories to high-quality males. In contrast, if the quality of the male’s resources (e.g., territories or nest sites) is of critical importance for female reproduction but not correlated with male condition, females may face a decision between selecting high-quality males or high-quality resources (Candolin & Voigt, 2001).

In the cichlid fish *Tropheus moorii*, an endemic of Lake Tanganyika, both intrinsic male traits and territory characteristics are plausibly used as mate choice cues by females. Consequently, female preferences could directly drive the evolution of male traits and could reinforce the selection pressure imposed by male–male competition for territories. Many populations in the genus *Tropheus* are conspicuously colored, and there is rich geographic color pattern variation within species (Konings, 2013). Cichlid color pattern diversification is often linked to male–male competition and female choice (Maan & Sefc, 2013), but the social system of *Tropheus*, with both male and female territoriality, also puts demands on female competitive abilities in territorial contests. Intersexual contests for territories may require females to match males in their resource holding potential. Indeed, most populations of *Tropheus* are sexually monomorphic in body size and color patterns, and body color signals are used by both males and females to communicate social status and motivation to territorial competitors (Wickler, 1969; Sturmbauer & Dallinger, 1995). Sexual monomorphism is typically associated with only weak intrasexual selection (Avise et al., 2002). However, uniparental maternal mouthbrooding by *Tropheus* females entails a male-biased operational sex ratio, such that female choice can create considerable variance in male mating success (Sefc, 2008; Steinwender et al., 2012). If female mate choice selects for a particular trait in males, and if this trait is also relevant for territorial success, intersexual competition may subsequently select for the same trait in females and maintain sexual monomorphism. Accordingly, female preferences for male coloration may have been involved in the evolution of the conspicuous color patterns shown by many *Tropheus* populations as well as in the allopatric diversification of color patterns among populations.

*Tropheus* feed on epilithic algae from rock surfaces in the shallow littoral zone. The mating success of *Tropheus* males depends on the possession of a territory, which enables them to supply feeding opportunities to their mates. Although solitary female *Tropheus* defend their own feeding territories, the energy drawn from their resources apparently does not allow them to mature for spawning (Yanagisawa & Nishida, 1991), perhaps because of inferior territory quality or because of the demands of territory defense. Possibly as a consequence of this requirement for nutrition, the mating system of this uniparental maternal mouthbrooder involves a pair bond of up to 3 weeks before spawning. During this time, a female feeds intensively in her mate’s territory in preparation for spawning, while the male takes care of territory defense. After spawning, the female abandons the male for solitary mouthbrooding (Yanagisawa & Nishida, 1991). Single paternity of broods (Egger et al., 2006) suggests that unpaired males do not partake in reproduction. In a population in northern Lake Tanganyika (*Tropheus* sp. “black” at Bemba), the territories of paired males were characterized by prominent rocks and large rock surface areas. Rock area in the males’ territories was correlated to male body size, and female territories were smaller and contained smaller rock areas than male territories (Yanagisawa & Nishida, 1991). Together, this suggests competition among males for high-quality territories, which may be required to provide critical nutritional resources to the females.

**Fig. 1:**
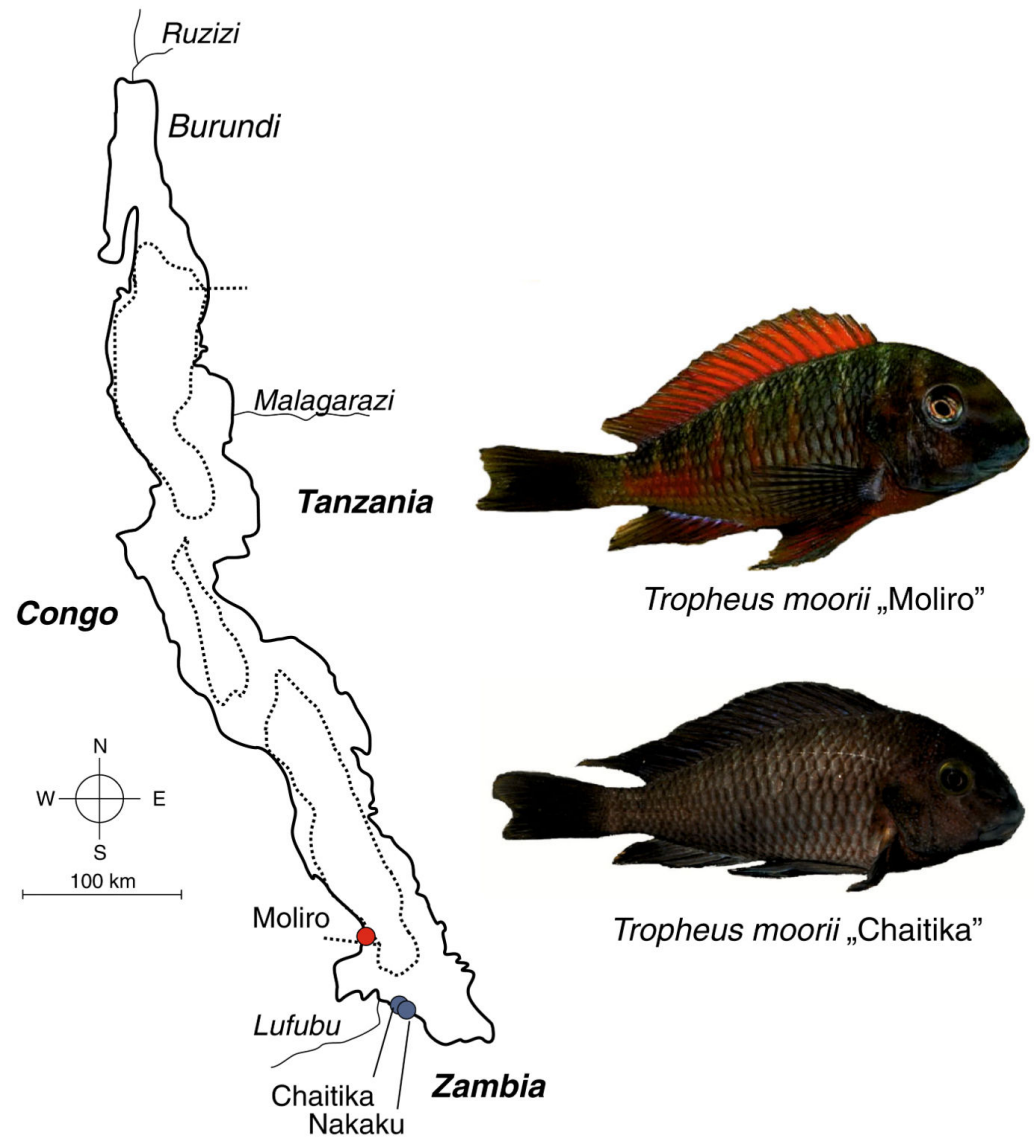
Lake Tanganyika and locations of the populations of red and bluish *Tropheus moorii* used in this work. The color pattern of the “Nakaku” *Tropheus* is very similar to the “Chaitika” shown in this figure

In the present study, two-way choice experiments were carried out to investigate the effects of male body size, coloration, territory size, and territory quality on female courtship preferences in a red-colored variant of *Tropheus moorii* present at Moliro (Fig. 1). Variation in male territory size and quality was ensured by placing two different males in either differently sized or differently furnished compartments. Because previous experiments on within-population mate choice in *Tropheus* suggested a correlation between male courtship quiver effort and female preference (Steinwender et al., 2012), we also scored male courtship activity and included it as covariate in the analyses.

Mate preferences in experimental encounters of differently colored *Tropheus* populations vary from random to strictly assortative (Egger et al., 2010; Zoppoth et al., 2013; Sefc et al., 2014, in review). One example of this is where females of the red-colored Moliro population have been shown to discriminate strictly against the blue-colored males from the Chaitika and Nakaku populations (Egger et al., 2008; Fig. 1), although cross-population offspring produced in no-choice situations is viable and fertile (Sefc, pers. observation). Genetic data have implied ancient hybridization between the two currently allopatric color variants, which probably occurred during secondary contact in the course of historic lake level fluctuations (Egger et al., 2007). In secondary contact, characteristics of the territories owned by different males may interfere with assortative mate preferences (Dijkstra et al., 2008). To test whether female preferences for superior territories, which were detected in the within-population experiment of the present study, also play a role in between-population mate choice and could override preferences for own males and induce mating between color morphs, we conducted another two-way female choice experiment, in which males of the divergent color variant were placed into the better furnished compartment.

## Materials and methods

All fish used in the experiments were wild caught and imported by an ornamental fish trader. Fish were held in large mixed-sex groups prior to and between experiments. At the beginning of each experiment, fish were moved into individual tanks (60 × 30 × 35 and 60 × 60 × 35 cm) or into individual compartments within a large tank (37.5 × 35.5 cm, separated by mesh partitions). All tanks were equipped with internal box filters, held at a temperature of 24–27 °C, and illuminated in a 12 h:12 h light:dark cycle. Fish were fed twice a day with a mixture of flakes and pellets occasionally supplemented with shrimps.

**Fig. 2:**
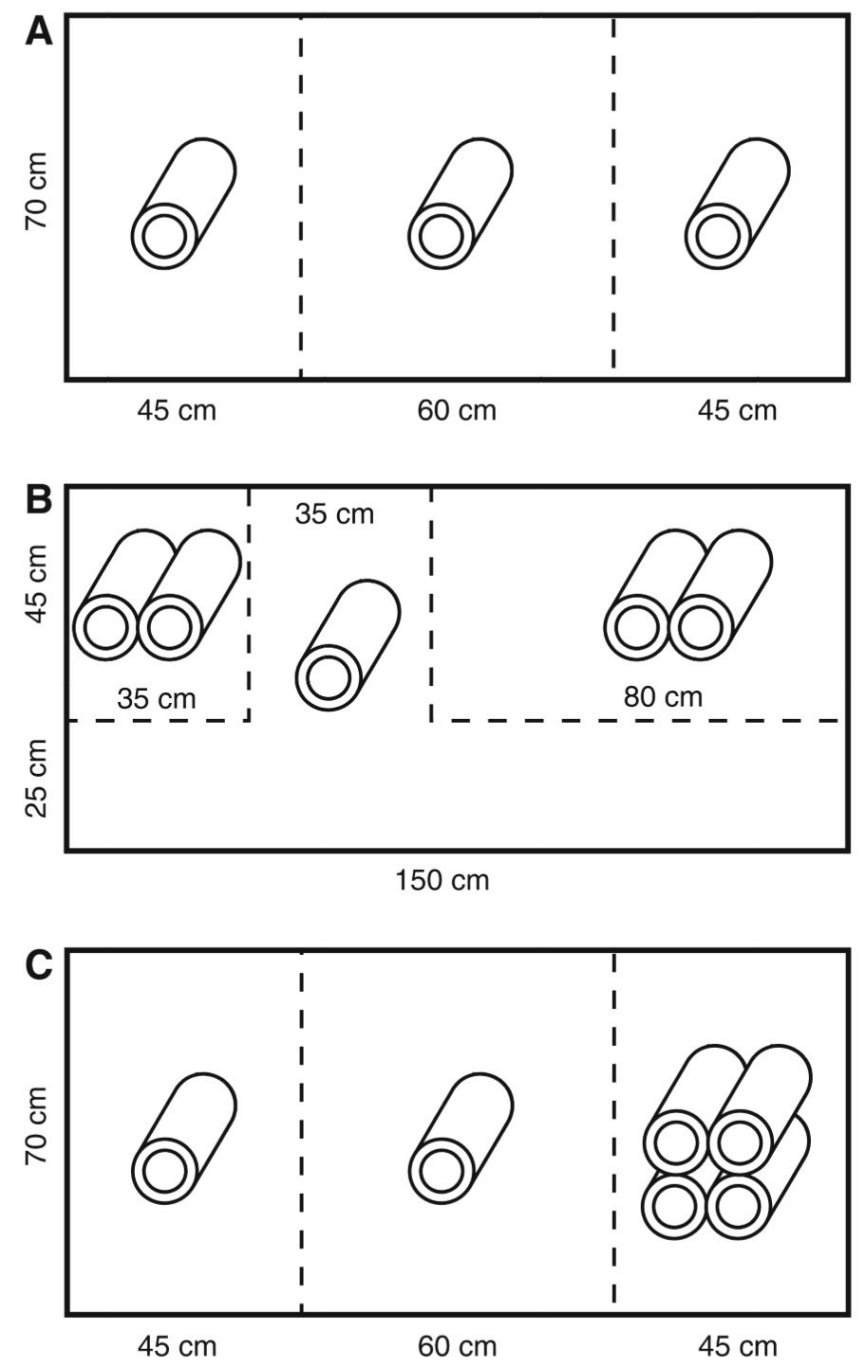
Dimensions and furnishing of compartments in the mate choice tanks. **A** experiment 1, **B** experiment 2, **C** experiments 3 and 4

Experimental tanks were divided into three compartments by mesh partitions and furnished with hollow brick cylinders as shown in Fig. 2. Each tank was stocked with a female in the center compartment and one stimulus male in each of the outer compartments. Weight and standard length (SL) of experimental fish were determined prior to each trial. Each trial consisted of an acclimatization and a sequential access phase (Egger et al., 2008). During the acclimatization period of 4–5 days (4 days in experiments 3 and 4), males and females remained separated by mesh partitions but could communicate by visual, olfactory, and acoustic signals. Next, females were allowed to interact freely with each of the two stimulus males at a time in “sequential access” sessions. To this aim, the partition between the female and one of the males was removed, while an opaque board was placed between the female compartment and that of the other male. An access session lasted for 30 min after the first interaction had occurred. Afterward, the male and female were separated again. After an interval of at least 2 h, the partition between the female and the other male was removed. Each trial consisted of 4–5 access sessions per stimulus male (4 in experiments 3 and 4). Two sessions per day (one session with each male) were carried out on consecutive days. The order of access (morning or afternoon session) was assigned randomly to the two males in a trial but remained the same across all days of a trial.

The rationale for using sequential access sessions in the mate choice experiments is as follows (see also Egger et al., 2008). In a previous experiment with *Tropheus*, the time spent near a male’s compartment and interactions at the compartment partitions were not related to mate preferences (Egger et al., 2008), making it necessary to observe unconstrained interactions between a pair. Because male and female *Tropheus* do not differ in body size, it is not possible to design compartment partitions, which would allow free movement of the focal females while retaining the males in their respective compartments (e.g., Plenderleith et al., 2005). If the partitions to both males were removed at the same time, male–male competition would interfere with the mate choice trial.

Fish were observed continuously during the access sessions, and courtship behavior was scored with EthoLog 2.2 (Ottoni, 2000). The recorded behaviors included quivering by males, quivering by females, and T-positions, in which one of the pair quivers and the other nuzzles the genital papilla of the partner (Nelissen, 1976). Male courtship effort was represented by male quiver rate (events per minute). Female responses to males were quantified by the summed rates (events per minute) of the female quivers and participation in T-positions.

### Experiment 1—effects of body size and dorsal fin color on within-population mate preferences

In this experiment, *T. moori* “Moliro” females were presented with two stimulus males of *T. moori* “Moliro”, which were placed in equally sized and furnished compartments (Fig. 2A). Each female was tested only once (*n* = 17). Individual males (*n* = 15) were used in 1–3 trials (mean 2.3) but always paired with a different male. Males were photographed for analysis of dorsal fin coloration after the mate choice trials were completed. The anterior part of the dorsal fin of this population is conspicuously red (Fig. 1). We only scored fin coloration and not body coloration, because the size and hue of skin color patches can change rapidly in response to stimuli such as handling, whereas the extent of red on the dorsal fin remains stable and is therefore better suited for analysis. For photography, the focal male was placed into a box made of transparent acrylic glass (15 × 15 × 50 cm), which was open on one side. The open front of the box was placed against the wall of the fish tank. A female was put into another box next to the male in order to stimulate males to display their dorsal fins. Photographs were taken with a digital camera (Olympus E-300 with sensor 8.0 megapixel; macro-lens Olympus Digital ED 50 mm 1:2) and standardized camera settings (one-touch white balance, aperture F/4.5; exposure time: 1/500 s; ISO: 400). Two light sources (Gun-Lux Typ 1001; 1000 W) were positioned left and right of the camera. Dorsal fins were cropped from the photographs, and the redscore (the extent of red coloration in % of total fin area) was determined in SigmaScan Pro 5.0 by counting the number of pixels with hue values from 0 to 30 and saturation from 70 to 100.

A linear model was used to test the effects of dorsal fin color, male body size, and male quiver rates on female preferences. For each trial, the male with the larger redscore was defined as “male1” and the relative differences between male traits were calculated as follows: relative body size difference RSD = (SL_male1_ − SL_male2_)/(SL_male1_ + SL_male2_); relative difference in dorsal fin redscore = (redscore_male1_ − redscore_male2_)/(redscore_male1_ + redscore_male2_); relative difference in male quiver activity RQD = (quiver rate_male1_ − quiver rate_male2_)/(quiver rate_male1_ + quiver rate_male2_). Relative differences in female courtship were expressed as RCD = (courtship rate_male1_ − courtship rate_male2_)/(courtship rate_male1_ + courtship rate_male2_), where courtship consists of female quivers and participation in T-positions.

A generalized linear mixed model (GLMM) with male and female identities as random factors was used to test whether male quiver activity was related to dorsal fin redscores and to male standard length. As the response variable (male quivers) was scored as counts, a negative binomial distribution (family “nbinom” in the R package glmmADMB) was used in R vs. 3.0.0 (R Development Core Team 2013). Significance was tested by a likelihood ratio test (LRT) comparing the models including dorsal fin redscore and male size with models excluding the factors.

### Experiment 2—effect of the size of male territory on within-population mate preferences

In this experiment, the compartments of the two *T. moori* “Moliro” stimulus males differed in size. The setup of the tanks (Fig. 2B) allowed *T. moori* “Moliro” females to assess the dimensions of the males’ compartments by swimming along the width and breadth of each compartment. In each trial, females were observed during the acclimatization phase to ensure that they indeed swam along each male’s compartment. Within each experimental tank, the sides (left, right), at which the large and small territories were set up, were switched between trials. Eighteen different females were tested with nine pairs of males (18 males in total). Each pair of males was used twice, as each male was placed in the small compartment in one trial and in the large compartment in the other trial (and vice versa for the second male of the pair).

Variables describing the relative differences between alternative males in body size, male quiver rates, and female courtship rates were calculated as described for experiment 1. “Male1” was defined as the male in the large compartment. A linear model with RCD as response variable and RSD and RQD as predictor variables tested the effects of territory size, male size, and quiver rate on female preferences. If male territory size had an effect on female preferences, the model intercept would deviate significantly from zero.

A GLMM tested whether male quiver activity differed between treatments (large and small compartments). Male and female identities were included as random factors. As the response variable (male quivers) was scored as counts, a negative binomial distribution (family “nbinom” in the R package glmmADMB) was used. A LRT was used to establish significance.

### Experiment 3—effect of the quality of male territory on within-population mate preferences

Here, the compartments of the two stimulus *T. moori* “Moliro” males were of equal size, but furnished with different numbers of hollow cylindrical bricks (Fig. 2C). In their natural territories, rock surfaces provide feeding grounds for *Tropheus*, which feed on epilithic algae, and caves serve as hiding places (Yanagisawa & Nishida, 1991). By providing both, the hollow brick cylinders are therefore expected to contribute to the attractiveness of a territory, and we consider the compartment with the larger brick structure to represent a territory superior in quality to the compartment with the smaller brick structure. Each *T. moori* “Moliro” female was tested only once (*n* = 12). Individual males (*n* = 14) were used in 1–2 trials (mean 1.7) but always paired with a different male. Males used in more than one trial received both the high-quality and low-quality treatment. Within each experimental tank, the larger and the smaller brick structure switched sides between trials.

Variables describing the relative differences between alternative males in body size, male quiver rates, and female courtship rates were calculated as described for experiment 1. “Male1” was defined as the male in the compartment with the larger brick structure. As in experiment 2, a linear model estimated the effect of territory quality, male size, and quiver rate on female preferences. If the quality of the territory had an effect on female preferences, the model intercept would deviate significantly from zero.

The effect of treatment (high- and low-quality territory) on male quiver rates was tested in a GLMM analogous to experiment 2. Additionally, to examine whether the slopes of the relationship between female courtship and male quiver rates differed between treatments, a LRT of GLMMs with and without the interaction term (male quiver rate × territory quality) was conducted. Male and female identities were included as random factors. As the response variable (female courtship) was scored as counts, a negative binomial distribution (family “nbinom1” in the R package glmmADMB) was used.

### Experiment 4—effect of territory quality on assortative mating between color variants

In this experiment, we tested whether known preferences of *T. moori* “Moliro” females for males of their own population over those from allopatric populations (Egger et al., 2008) would be reduced when the allopatric males possessed the superior territory. The experiment used ten red *T. moori* “Moliro” females, ten red *T. moori* “Moliro” males, eight bluish *T. moori* “Nakaku” males, and one bluish *T. moori* “Chaitika” male, which participated in two trials. Each female was tested once against a pair of males.

The compartments for males and females were furnished as in experiment 3 (Fig. 2C). The bluish males were placed in the compartment with the larger brick structure and the red males in the compartment with the smaller structure. The frequencies of courtship interactions between males and females in the sequential access phase were tallied as in the other experiments. In the previous experiment of Egger et al. (2008), the clear preferences of red females for red males had allowed us to score trial outcomes as a binary variable (preference for one or the other male). Similarly, in most trials of the present experiment, courtship occurred only between the female and one of the two alternative males. Therefore, we again scored female preferences as a binary response (see “Results” section for details) and combined the data of the previous and the present experiment into a logistic regression to test the effect of “experiment” (i.e., equal-compartment experiment, different-compartment experiment) on the assortative preferences of red females. Firth’s bias reduced logistic regression implemented in the R package logistf (Heinze & Schemper, 2002) was applied in order to account for the fact that all red females in the equal-compartment experiment had preferred the red male (i.e., the perfect correlation between response and treatment).

## Results

### Within-population mate choice

#### Experiment 1—body size and redness of dorsal fin

**Fig. 3:**
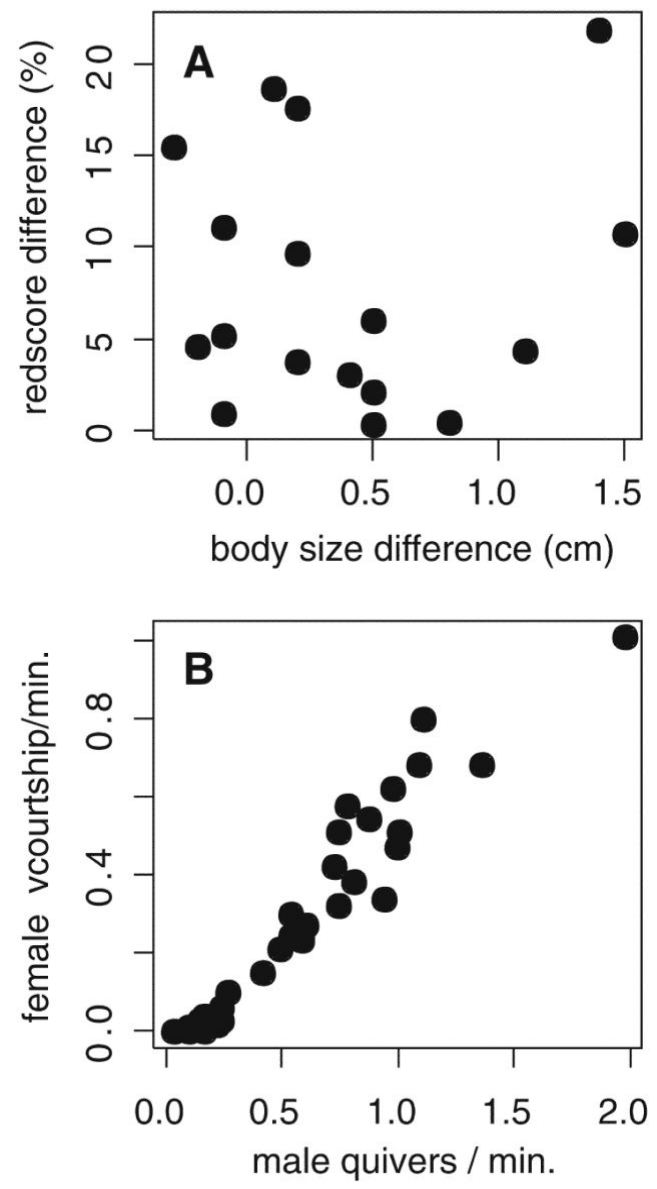
**A** Differences in dorsal fin redscore between paired males in experiment 1 plotted against their body size differences. **B** Rates of female courtship plotted against male quiver rates (experiment 1)

The measures of red-colored area in the dorsal fins of males ranged from 3.3 to 25.2% of total fin area (mean ± SD = 17.9 ± 6.57%). Body sizes of males ranged from 8.0 to 10.5 cm SL (mean ± SD = 9.5 ± 0.58 cm). Dorsal fin redness was not correlated with male size, when the smallest and least red individual was removed from the analysis (Pearson *r* = 0.24, df = 12, *P* = 0.41). Male quiver rates displayed in the mate choice trials were independent of dorsal fin redscores (LRT: χ^2^ = 0.376, df = 1, *P* = 0.54) and body size (LRT: χ^2^ = 0.010, df = 1, *P* = 0.92). Males paired in trials differed in their standard length by 0.1–1.5 cm and in the red dorsal fin area by 0.4–21.9%. Within-pair differences in body size and red dorsal fin area were not correlated (Fig. 3A). Rates of female courtship, i.e., quivering and participation in T-positions, increased with male quiver rates (Fig. 3B). Relative differences in dorsal fin redscores and body size between alternative males had no effect on female preferences; the only significant factor in the model was the relative difference in male quiver activity (Table 1).

#### Experiment 2—territory size

**Fig. 4:**
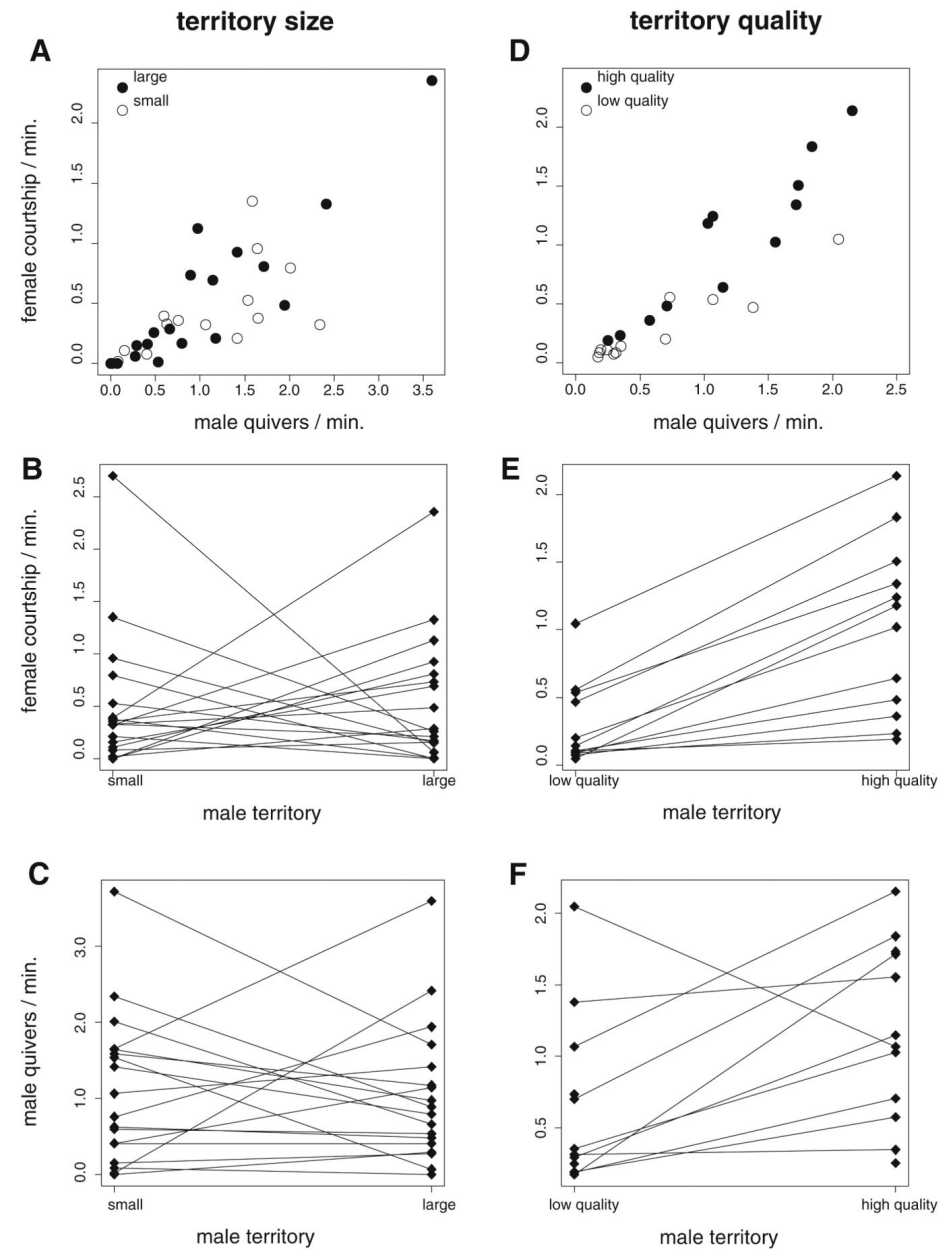
Female and male courtship in experiments testing the effects of territory size (experiment 2) and quality (experiment 3). **A, D** Rates of female courtship plotted against male quiver rates. **B, E** Female courtship with males in the alternative treatments. *Lines* connect the values obtained from the same female. **C, F** Male quiver activity in the alternative treatments. *Lines* connect values scored from the same male. In experiment 3, some males were tested in only one treatment

Rates of female courtship co-varied with male quiver rates. The slope of this relationship was independent of male territory size (Fig. 4A). Neither male territory size nor relative body size differences between the two males in a trial affected female preferences; the only significant factor in the model was the relative difference in male quiver activity (Table 2; Fig. 4B). Quiver rates of individual males were either similar in small and large territories or varied between trials independent of territory size (Fig. 4C). Across males, quiver activity did not differ between the small-territory and the large-territory treatment (LRT: χ^2^ = 0.032, df = 1, *P* = 0.86).

#### Experiment 3—territory quality

Both territory quality and differences in male quiver rates were related to female preferences, whereas differences in male body size had no effect (Table 3; Fig. 4E). Males displayed higher quiver activity in the superior territory than in the inferior territory (Fig. 4F; LRT: χ^2^ = 16.922, df = 1, *P* = 0.00004). Female courtship rates increased more strongly with the quiver rates of males in superior territories than of males in inferior territories (Fig. 4D), but the interaction term between male quiver rates and territory quality was not significant (LRT: χ^2^ = 0.61, df = 1, *P* = 0.43).

### Between-population mate choice

#### Experiment 4—effect of territory quality on assortative female preferences

In the present experiment, eight of the ten red *T. moori* “Moliro” females clearly preferred the red *T. moori* “Moliro” male and rejected the bluish allopatric male, whereas two females courted with both males, such that no preference could be inferred. In all but one trial with positive assortative outcomes, bluish males showed no quiver activity toward red females, and red females courted exclusively with red males. As we were interested in whether or not a superior territory would lower the rejection rate of bluish males by red females in comparison to the Egger et al. (2008) experiment, we scored trial outcomes as 0, when the female clearly rejected the bluish male (i.e., in all trials of Egger et al., and in eight trials of the present experiment), and as 1 for the two trials in which females also courted with the bluish male. A logistic regression showed that in the present experiment, bluish males were more likely to be rejected by red females than to be courted, but the difference was not significant (*P* = 0.06; Table 4). The rejection rate suffered by bluish males did not differ between the experiments with equally and differently furnished male compartments (Table 4).

## Discussion

In our experiments, female mate preferences were unaffected by differences in body size, dorsal fin redness, and territory size between the alternative males. In contrast, the quality of the males’ territories had a significant effect on within-population mate preferences, as females always displayed higher courtship activity with the male in the compartment furnished with a larger brick structure. Discrimination according to solid surface area but not according to compartment size is consistent with the putative role of the male territory as a source of epilithic algae for the female in preparation for spawning (Yanagisawa & Nishida, 1991). In addition to food, territories also offer shelter and protection from predators, as *Tropheus* hide in rock crevices and in the cavities between layers of rocks and stones. Shelter quality may therefore be another criterion by which females discriminate among male territories.

Alternatively, females might be able to draw the required resources from any male territory but use territory characteristics as indicators of male quality. Male–male competition links territory quality to male condition, such that preferences for superior territories also could ensure indirect benefits obtained through increased offspring fitness. Additionally, the dependency of competitive success on body size (Odreitz, 2012) suggests that in the field, territorial contests will result in a correlation between territory quality and male body size (Yanagisawa & Nishida, 1991). Female *Tropheus* could presumably benefit from large mates, because large males suffer fewer and less successful attacks (Kohda & Yanagisawa, 1992) and can therefore provide better conditions for female boost-feeding than small males. In the shell-breeding cichlid *Lamprologus callipterus*, for in stance, female preferences for large males reflect the risk of expulsion and infanticide met by females mated to small males (Maan & Taborsky, 2008). However, if female choice was primarily for large or healthy males, it seems surprising that male size and color had no influence on female preferences in experiments with invariable male territories. Male body size can be directly assessed by females, and red carotenoid-based coloration may be an honest indicator of male quality (Sefc et al., 2014). In the haplochromine cichlid *Pundamilia nyererei*, male redness is correlated to various correlates of health and condition (Maan et al., 2006; Dijkstra et al., 2007; Dijkstra et al., 2011), and preferences of *P. nyererei* females for males with high redscores over less red males therefore may select for heritable fitness (Maan et al., 2004). Male size or redness affects female preferences in a number of other fish species (e.g., Milinski & Bakker, 1990; Ptacek & Travis, 1997; Rosenthal & Evans, 1998; Basolo, 2004; Hudman & Gotelli, 2007; Karino & Urano, 2008; Passos et al., 2013), but exceptions exist (e.g., Kodric-Brown, 1983; Reichard et al., 2005; Amorim et al., 2013). Greater differences in size and coloration between alternative *Tropheus* males than those in our experiment might possibly produce an effect but would not correspond to the natural variation among potential mates (Yanagisawa & Nishida, 1991; Kohda & Yanagisawa, 1992), which is represented by the wild-caught adults in our experiment.

While our present experiments identified no effects of male body traits on female preferences, a previous two-way choice experiment with males in identical compartments had suggested that females use consistent, but unknown, criteria to discriminate between alternative males (Steinwender et al., 2012). In that experiment, four pairs of red-colored *Tropheus moorii* “Chimba” males were presented to females of their own population. With one exception, females shared preferences for the same males and the preferred males displayed higher courtship activity than their rejected counterparts (Steinwender et al., 2012). The experiment of Steinwender et al. (2012) was not designed to identify male traits which could predict female choice, but the congruent female preferences suggested that mate choice was not based on compatibility between male and female genotypes (Neff & Pitcher, 2005), which if applicable could have helped to explain the decoupling of female preferences from male body traits in the present study.

Could male behavior inform females about male quality and be used as mate choice cue? Energetically demanding courtship may honestly indicate male condition (Knapp & Kovach, 1991) and fertility (Weir & Grant, 2010). Moreover, sounds produced during the quiver display of cichlid males (including *Tropheus*, Nelissen, 1978) attract female attention and possibly affect female choice (Verzijden et al., 2010; Maruska et al., 2012). However, the correlations between female preferences and male courtship vigor, which have been observed in a wide range of animal taxa, do not necessarily imply that female preferences are determined by male courtship. Rather, in a number of studies, males were found to adjust their courtship efforts in response to female behavior (Collins, 1994; Rutstein et al., 2007; Takahashi et al., 2008; Royle & Pike, 2010; Rodríguez et al., 2012; Kahn et al., 2013). In the present study, male quiver rates were positively correlated to female courtship in all experiments and regardless of treatment. Importantly, in the experiment presenting males in differently furnished compartments, the courtship effort of individual males varied between treatments. Although the allocation of males to inferior and superior compartments was independent of male quality, males in superior compartments quivered more than males in inferior compartments. Moreover, most of the individual males, which were tested in both compartments, showed higher quiver rates in the superior compartment (Fig. 4F). Therefore, at least in the territory quality-experiment, male quiver rates do not seem to reflect intrinsic male qualities. In contrast, intense courtship by males in superior compartments may follow from female preferences for high-quality territories, either as a direct response to female behavior or, if males were able to gage the attractiveness of their resource against that of their opponent, because males adjusted their courtship to the likelihood of being successful (Kahn et al., 2013). Correlations between manipulated male attractiveness and male courtship intensity were observed in other species as well (e.g., zebrafinch: Royle & Pike, 2010; sticklebacks: Morrell et al., 2012; damselfish: Snekser et al., 2009), whereas in bitterling fish, experimental resource allocation did not influence male behavior (Candolin & Reynolds, 2001). Alternatively, territory quality might affect male courtship activity independent of female behavior, if males can afford more conspicuous courtship when they occupy territories which provide sufficient shelter from predators. In our experiment, riskier behavior in the high-quality compartment could have been stimulated by the extra hiding spaces provided by the double layer of bricks (Fig. 2C). A strong correlation between the number of caves defended by males and their courtship activities was also observed in a field study of a cichlid species in Lake Malawi (Markert & Arnegard, 2007) and could represent behavioral responses to differences in predator protection capacity among territories.

While territory quality had a distinct effect on within-population female preferences in the red-colored *Tropheus moori* “Moliro,” it did not significantly affect assortative mating between red and bluish *Tropheus*. Although bluish males occupied the superior compartment in the two-way choice experiment, red females preferred their own males in inferior compartments over bluish males in the majority of trials. Assortative preferences of males could contribute to maintaining assortative mate choice, if females attracted to the bluish males’ superior territories failed to elicit a courtship response from the bluish males. Indeed, bluish males displayed little courtship when red females inspected their compartment. In between-population male mate choice tests, red *Tropheus* “Moliro” males expressed assortative courtship preferences that were independent of female courtship (Zoppoth et al., 2013), which raises the possibility that males of other color variants also discriminate against differently colored females. Moreover, we have previously observed that red males dominated over bluish males in staged territorial contests (H. Zimmermann, unpublished data). The bluish males in our experiment could have been intimidated by red females, whom they perhaps perceived as territorial competitors rather than potential mates.

Our results contrast with those obtained in two closely related cichlid species from Lake Victoria, *Pundamilia nyererei* (red males), and its sympatric congener, *P. pundamilia* (blue males). In these sexually dimorphic cichlids, males of both species court the cryptically colored con- and heterospecific females equally (Haesler & Seehausen, 2005), yet females of both species prefer conspecific males (Seehausen & van Alphen, 1998). However, when the red *P. nyererei* females were given the choice between red males with small and blue males with large shelters in their respective compartments, they slightly preferred blue males over red males, while both males still displayed equal courtship activity (Dijkstra et al., 2008). In *Tropheus*, premating reproductive isolation between the allopatric red and bluish populations has apparently advanced to a degree that male territory characteristics do no longer have a substantial influence on mate choice. As female assortative preferences have been shown to be stronger in the red-colored “Moliro” population than in other *Tropheus* color variants (Egger et al., 2010), the result of the current study does not refute the possibility that male territory quality can modify mate choice in color variants with weaker assortative preferences.

In conclusion, our data suggest that within- and between-population mate preferences of red-colored *Tropheus* females depend on different cues. Within the population, female choice is strongly influenced by preferences for high-quality territories, whereas between color variants, population-assortative preferences prevail over territory cues. Male characteristics influencing female preferences in within-population mate choice remain elusive. Female preferences covaried with male courtship effort, but the observation that courtship rates of individual males were contingent on the quality of their territory suggests that courtship effort is not directly dependent on male quality.

## Figures and Tables

**Table 1:** Results of the general linear model testing the effects of male dorsal fin coloration, male quiver activity, and male body size on female preferences (experiment 1)

Fixed effect	Value	SE	*t*	*P*
Intercept	0.0600	0.0997	0.600	0.559
Male quiver rate	1.3566	0.1348	10.066	1.67 × 10^−7^
Dorsal fin redscore	−0.0772	0.2395	−0.302	0.768
Male body size	−0.0533	2.3930	−0.22	0.983

**Table 2:** Results of the general linear model testing the effects of male territory size, male quiver activity and male body size on female preferences (experiment 2)

Fixed effect	Value	SE	*t*	*P*
Intercept	−0.0147	0.0564	−0.261	0.798
Male quiver rate	1.075	0.0876	12.279	3.16 × 10^−9^
Male body size	−0.0856	1.8562	−0.046	0.964

The non-significant intercept value implies that female preferences were independent of the sizes of the males’ territories

**Table 3:** Results of the general linear model testing the effects of male territory quality, male quiver activity and male body size on female preferences (experiment 3)

Fixed effect	Value	SE	*t*	*P*
Intercept	0.3121	0.0538	5.805	0.000258
Male quiver rate	0.7922	0.1385	5.721	0.000287
Male body size	−0.3613	0.8610	−0.420	0.6846

The significantly positive intercept value implies female preferences for males in superior territories

**Table 4:** Results of the logistic regression testing the effect of territory quality on the rejection of bluish males by red female (experiment 4)

Fixed effect	Value	SE	χ ^2^	*P*
Intercept	−1.2238	0.7546	3.458	0.063
Experiment (Egger et al., 2008)	−1.4843	1.7342	1.042	0.307

Data on mating preferences in the Egger et al. (2008) experiment and the present experiment were combined for this analysis. The negative intercept value indicates that in the present experiment, bluish males were more likely to be rejected by red females than to be courted. The non-significant effect of “Experiment” indicates that the rejection rate of bluish males did not differ between the two experiments
